# Plumbagin Alleviates Capillarization of Hepatic Sinusoids In Vitro by Downregulating ET-1, VEGF, LN, and Type IV Collagen

**DOI:** 10.1155/2017/5603216

**Published:** 2017-07-09

**Authors:** Guiyu Li, Yue Peng, Tiejian Zhao, Jiyong Lin, Xuelin Duan, Yanfei Wei, Jing Ma

**Affiliations:** ^1^Graduate School, Guangxi University of Chinese Medicine, No. 13 Wuhe Road, Nanning, Guangxi 530200, China; ^2^Department of Physiology, Faculty of Basic Medicine, Guangxi University of Chinese Medicine, No. 13 Wuhe Road, Nanning, Guangxi 530200, China

## Abstract

Critical roles for liver sinusoidal endothelial cells (LSECs) in liver fibrosis have been demonstrated, while little is known regarding the underlying molecular mechanisms of drugs delivered to the LSECs. Our previous study revealed that plumbagin plays an antifibrotic role in liver fibrosis. In this study, we investigated whether plumbagin alleviates capillarization of hepatic sinusoids by downregulating endothelin-1 (ET-1), vascular endothelial growth factor (VEGF), laminin (LN), and type IV collagen on leptin-stimulated LSECs. We found that normal LSECs had mostly open fenestrae and no organized basement membrane. Leptin-stimulated LSECs showed the formation of a continuous basement membrane with few open fenestrae, which were the features of capillarization. Expression of ET-1, VEGF, LN, and type IV collagen was enhanced in leptin-stimulated LSECs. Plumbagin was used to treat leptin-stimulated LSECs. The sizes and numbers of open fenestrae were markedly decreased, and no basement membrane production was found after plumbagin administration. Plumbagin decreased the levels of ET-1, VEGF, LN, and type IV collagen in leptin-stimulated LSECs. Plumbagin promoted downregulation of ET-1, VEGF, LN, and type IV collagen mRNA. Altogether, our data reveal that plumbagin reverses capillarization of hepatic sinusoids by downregulation of ET-1, VEGF, LN, and type IV collagen.

## 1. Introduction

Inflammation, chronic viral hepatitis, and liver injury are considered to be canonical causes of hepatic fibrogenesis. Liver injury leads to loss of liver sinusoidal fenestration, which may promote fibrosis by sinusoidal thrombi [1]. The crucial roles of liver sinusoidal endothelial cells (LSECs) on the initiation and progression of chronic liver diseases have been illustrated. LSECs are highly specialized endothelial cells. They have a discontinuous architecture demonstrating the formation of fenestrae [2–4]. LSECs are a primary regulator of small molecule exchange between sinusoids and hepatic parenchyma due to open fenestrae with small diameters that allow for tight control and enhancement of metabolic interchange [5]. Oxygen, nutrients, hormones, and inflammatory factors from the blood spread through the sinusoidal lumen. Hepatic diseases are associated with the distribution of inflammatory infiltrates, toxins, and neoplasm from the sinusoids to the liver. LSECs constitute a major cell type of the hepatic sinusoid [6]. They play a key role in inflammation by upregulating the junction adhesion molecules JAM-B and JAM-C during liver fibrosis [7].

While LSECs have large amounts of open fenestrae, they lack a continuous basement membrane [8]. The space of Disse is localized among the LSECs and neighboring hepatocytes due to the loss of the basement membrane between the two [6]. Nevertheless, the loss of fenestrae accompanied by a decrease in the diameter of the fenestrae is termed defenestration and results in the formation of a continuous basement membrane [9, 10]. This process is termed the “capillarization” of the hepatic sinusoid [11] and is often seen in LSECs. The sinusoidal capillarization of the LSEC phenotype precedes obvious inflammation in the early stages and promotes liver fibrosis progression [12]. Evaluating LSECs is difficult since there are few drugs delivered to LSECs.

Upregulation of endothelin-1 (ET-1), vascular endothelial growth factor (VEGF), laminin (LN), and type IV collagen by LSECs during liver fibrosis has been reported [13–15]. VEGF plays a role in the differentiation of the LSEC phenotype [9]. ET-1 activates myofibroblasts, which can contribute to fibrosis [16]. ET-1 may influence hepatic microcirculation facilitating capillarization [17]. LN and type IV collagen are major components of the ECM and basement membrane. During hepatic fibrogenesis, these excessively deposit in the liver, resulting in the formation of a basement membrane [18, 19]. All of the above results suggest that ET-1, VEGF, LN, and type IV collagen participate in liver fibrosis.

In our previous study, we found a drug, plumbagin, that suppressed hepatic stellate cell (HSC) activation and contributed to the activation of HSC apoptosis during liver fibrosis. Plumbagin, a Chinese herbal medicine, promotes blood circulation for the removal of blood stasis. However, the effects of plumbagin on LSECs remain undetermined.

We hypothesized that plumbagin would reverse liver fibrosis in another way, through its accumulation in LSEC and subsequent amelioration of phenotype changes and dysfunction via decreasing profibrogenic factors, including ET-1 and VEGF, as well as reducing components of basement membrane LN and type IV collagen. In this study, we delivered plumbagin to leptin-stimulated LSECs. Our purpose was to illustrate that the effects of plumbagin on alleviating phenotype changes and dysfunction in the leptin-stimulated LSEC were achieved via the decreased expression of ET-1, VEGF, LN, and type IV collagen, effectively alleviating the mechanism causative of hepatic fibrosis.

## 2. Materials and Methods

### 2.1. Drugs and Reagents

Plumbagin was purchased from Sigma Chemical Company (St. Louis, MO, United States). Dimethylnitrosamine (DNM) was purchased from Dahao Chemical Company (Shantou, Guangdong, China). Dimethylsulfoxide (DMSO) was purchased from Solarbio Technology Company (Beijing, China). Dulbecco's modified Eagle medium (DMEM) and fetal bovine serum (FBS) were purchased from GIBCO.

### 2.2. Animals

The experiments were performed using 8-week-old male Sprague Dawley (SD) rats, which were obtained from the Medical Laboratory Animal Center of Guangxi Medical University, China (using number SCXK-Gui-2009-0002). The body weights of the rats were 150~180 g. At least 3 animals were needed for the experiment. The animal studies were performed in the Animal Research Center at Guangxi University of Chinese Medicine (Nanning, Guangxi) and approved by the Institutional Animal Ethics Committee of Guangxi University of Chinese Medicine, China. All authors ensured that all experiments were performed in accordance with the approved guidelines and regulations. All animals were maintained under standard conditions (22°C and 12 h light/dark cycles) for further experiments.

### 2.3. Cells Isolation and Culture

To obtain primary LSECs, male Sprague Dawley (SD) rats were used. Isolation of primary rat LSECs was performed as published [20]. Briefly, LSECs were isolated from the rats' livers through hepatic collagenase perfusion, differential speed centrifugation, and density gradient sedimentation in Percoll (Tiandz, Inc., Beijing, China) as well as cell selective adherence. Immunostaining with cytokeratin-19 antibody (Wuhan Boster Biological Technology, Ltd., Wuhan, China) was used to determine the purity of these cells [21]. Cells were identified by scanning electron microscopy and were cultured in DMEM containing 10% FBS. Suitable conditions, including moderate room temperature and a humidified atmosphere of 5% CO_2_, were used to maintain the cells. After reaching 80~90% confluency, the cells were cultured on six-well plates at a density of 1 × 10^5^ cells per well for 48 hours prior to experiments.

For maintaining LSECs phenotype after the isolation, on the one hand, the cells were cultured in DMEM containing 10% FBS and 10 ng/mL endothelial cell growth factor (ECGF, purchased from PeproTech, United States). On the other hand, primary cells seeded on coverslips coated with collagen were used for experiments and were rapidly fixed with glutaraldehyde for electron microscopy.

All authors ensured that the experiments protocols related to animals were approved by the Institutional Animal Ethics Committee of Guangxi University of Chinese Medicine. All experiments with animals were performed in accordance with the approved guidelines and regulations.

### 2.4. Induction of Activation

The cells were grown in six-well plates. To stimulate LSECs, 100 *μ*g/L leptin was added to the wells for 48 hours [22].

### 2.5. Experimental Groups and Treatment

The cells were divided into 3 groups: (1) vehicle control group without leptin or drug; (2) leptin group, or the leptin-stimulated LSECs model group, without any drug treatment, in which 100 *μ*g/L leptin was added to the wells to stimulate LSECs; (3) plumbagin + leptin-stimulated group with leptin for stimulating LSECs and plumbagin for cells treatment. Leptin (100 *μ*g/L) was added to the wells. The cells were then treated with two different doses (2 and 15 *μ*mol/L) of plumbagin for 48 hours.

### 2.6. Scanning Electron Microscopy

To investigate changes in LSEC fenestrae, we used scanning electron microscopy (SEM). Twenty-four hours after administration, all samples were prepared for SEM. The preparations were washed using phosphate-buffered solution (PBS) twice and then fixed rapidly with 2.5% glutaraldehyde for 1 hour. The preparations were washed with PBS twice, followed by 30%, 50%, 70%, and 80% ethanol at 4°C and 90%, 95%, and 100% ethanol at room temperature to dehydrate the prepared samples, all for 10 minutes. The preparations were then placed in 100% isoamyl acetate ester and dried. The preparations were placed on a metal stub. Platinum (15 nm) was used as the surface coating for the prepared samples. The samples were viewed with a VEGA3 scanning electron microscope (Hitachi S-4800, Tokyo, Japan).

### 2.7. Transmission Electron Microscopy

The isolated LSECs were rapidly immersed in 2.5% glutaraldehyde for 1 hour and then washed in PBS three times. Then, 1% OsO_4_ was added for 2 hours to fix the cells. The cells were then dehydrated via a graded acetone solution. Epoxy was used to embed the cells. Ultrathin sections were then passed through copper grids, and uranyl acetate and lead citrate were added to stain the sections. Finally, the sections were imaged using a transmission electron microscope (Hitachi H-7650, Tokyo, Japan). The sections were stained using uranyl acetate and lead citrate. Copper grids were used to maintain the ultrathin size of the sections.

### 2.8. Immunofluorescence Assays

To investigate the expression of ET-1, VEGF, LN, and type IV collagen in LSECs, cells on coverslips were washed with PBS three times for three minutes each time and then fixed with 4% poly-formaldehyde solution (5 ml per well). After 15 minutes, the cells were washed with PBS three times. Then, the cells were incubated in 0.5% Triton X-100 containing goat serum and incubated with anti-ET-1, anti-VEGF, anti-LN, and anti-type IV collagen and the corresponding Cy3-conjugated anti-IgG antibodies. Coverslips were mounted on glass slides with anti-fluorescence quenching sealing (Southern Biotech Company) mounting medium with DAPI (Beyotime, Biotech Company, Shanghai, China) and visualized using a fluorescence inverted microscope (Olympus, Japan).

### 2.9. ELISA

The levels of ET-1, VEGF, LN, and type IV collagen in LSECs were determined via ELISA using the corresponding assay kits (Elabscience) according to the manufacturer's instructions. Briefly, medium was added to ELISA plates coated with anti-rat ET-1, VEGF, type IV collagen, and LN antibody (Elabscience) and incubated at 37°C for 1.5 hours. Next, the corresponding biotinylated detection antibody was added to the plates and incubated at 37°C for 1 hour. This was followed by adding of avidin-conjugated horseradish-peroxidase (HRP) to the plates. Finally, chromogenic substrate was added to the plates. An ELISA microplate reader was used to detect levels of rat ET-1, VEGF, type IV collagen, and LN antibodies.

### 2.10. Quantitative Real-Time PCR (qPCR)

To investigate the levels of ET-1, VEGF, LN, and type IV collagen mRNA in LSECs, quantitative RT-PCR was performed. Total RNA was extracted from LSECs using a total RNA Extraction Kit (TIANGEN BIOTECH, Beijing, China). Then, total RNA reverse transcription into cDNA was performed using a RevertAid First Strand cDNA Synthesis Kit (Thermo Scientific). For qPCR, SYBR Green or Fluorescein qPCR Master Mix (2x) (Thermo Scientific, Waltham, USA) was used. Specific PCR primers sequences were as follows: ET-1 forward, 5′-TCCCGTGATCTTCTCTCTGC-3′, reverse, 5′-TGACCCAGATGATGTCCAGG-3′ (212 bp); VEGF forward, 5′-CGTCTACCAGCGCAGCTATTG-3′, reverse, 5′-CTCCAGGGCTTCATCATTGC-3′ (145 bp); type IV collagen forward, 5′-GGGTGATTGTGGTGGCTCTG-3′, reverse, 5′-CCTCGTGTCCCTTTCGTTCC-3′ (198 bp); LN forward, 5′-GACCCGTTCGGTTGTAAAT, reverse, 5′-GCCAGACTCCACCTCGTTA-3′ (288 bp); *β*-actin forward, 5′-CACGATGGAGGGGCCGGACTCATC-3′, reverse, 5′-TAAAGACCTCTATGCCAACACAGT-3′ (240 bp). Reactions were performed at 50°C for 2 minutes, 95°C for 10 minutes, 95°C for 30 seconds, and 60°C for 30 seconds. The process was performed for 40 cycles. Then, 2% agarose gels were used to separate the PCR products for observation. Additionally, for the detection, *β*-actin was used as an internal control. An ABI system (7900HT Fast Real-Time PCR) was used for data analysis.

### 2.11. Statistical Analysis

Data were analyzed using SPSS statistic 16.0 software and presented as the mean ± SD. ANOVA with Bonferroni posttests were used to evaluate significant differences between groups. *P* values < 0.05 were considered significant differences between independent groups.

## 3. Results

### 3.1. Defenestration of LSECs and Formation of Basement Membrane Are Induced by Leptin

**Figure 1:**
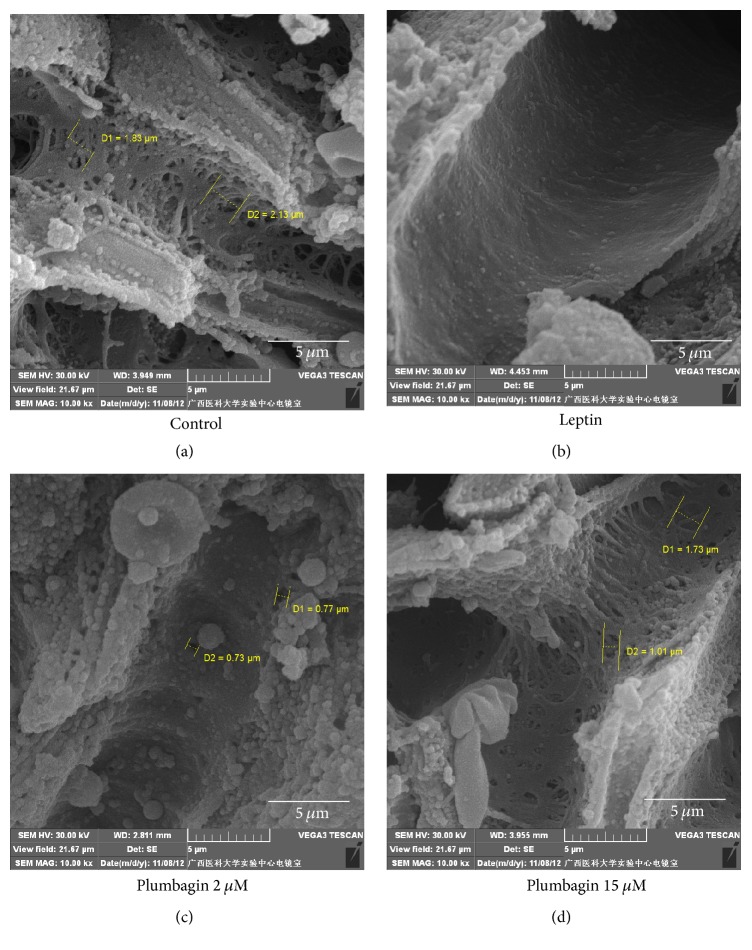
Plumbagin returned fenestrae loss in vitro study: the numbers and diameter of fenestrae were detected by scanning electron microscope examination. (a) Control group had large numbers of open fenestrae with 1.83–2.13 diameter. (b) There were no open fenestrae in the leptin-stimulated group. (c) Plumbagin with 2 *μ*mol/L increased the size and numbers of open fenestrae. The biggest size of open fenestrae in 2 *μ*mol/L plumbagin group was 0.77 *μ*m. (d) Plumbagin with 15 *μ*mol/L markedly increased the size (1.01–1.73 *μ*m) and numbers of open fenestrae. The effects of 15 *μ*mol/L plumbagin on increasing the size and numbers of open fenestrae were better than 2 *μ*mol/L. Scale bar, 5 *μ*m.

**Figure 2:**
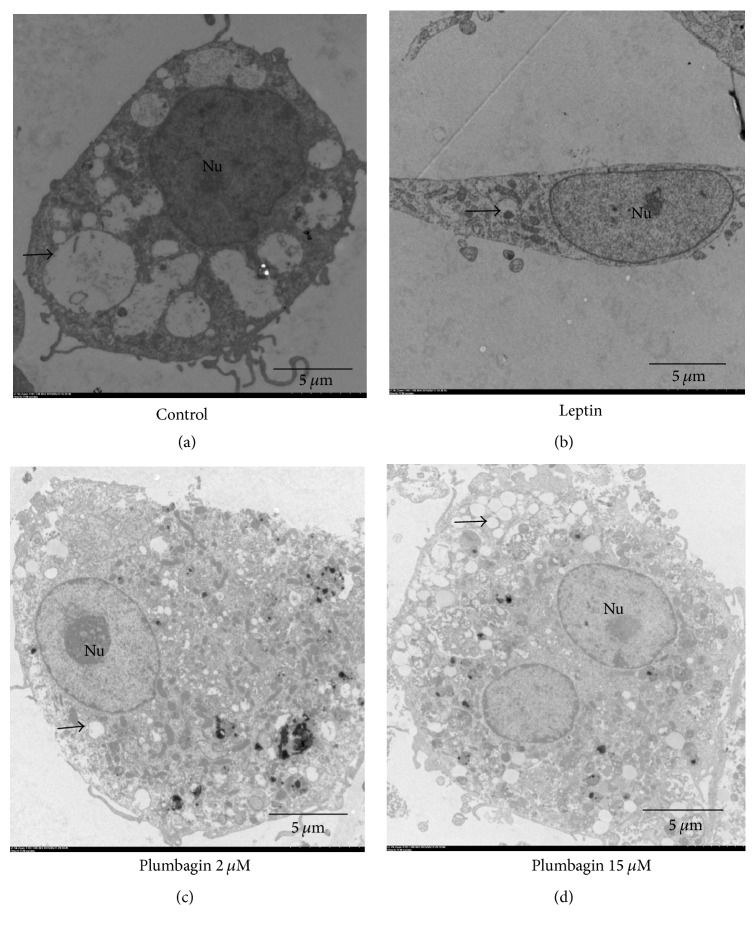
Plumbagin reverses capillarization of hepatic sinusoid, as seen by transmission electron microscopy. LSECs were stimulated via leptin and then treated with plumbagin (2, 15 *μ*mol/L) for 48 hours. Scale bar, 5 *μ*m. Arrows represent the fenestrae. Nu, nucleolus.

In normal LSECs, there were large amounts of open fenestrae and no continuous basement membrane. In contrast, continuous basement membranes were visible in the leptin-stimulated group. Few open fenestrae were observed in the leptin-stimulated group. All of the above results indicated that leptin mediated the defenestration of LSECs and the formation of a basement membrane, as shown by scanning electron microscope and transmission electron microscopies (Figures 1 and 2).

### 3.2. Plumbagin Ameliorates Defenestration of LSECs and Production of Basement Membrane

After 2 and 15 *μ*mol/L plumbagin administration, the open fenestrae were increased, and thin basement membrane-like structures were visible. Moreover, the sizes and numbers of open fenestrae were markedly increased, and no basement membrane production was observed after plumbagin administration according to scanning electron microscope (Figure 1) and transmission electron microscopies (Figure 2). The results indicated that plumbagin alleviated the defenestration of LSECs and the formation of a basement membrane.

### 3.3. Plumbagin Reduces Deposition of ET-1, VEGF, LN, and Type IV Collagen in LSECs

**Figure 3:**
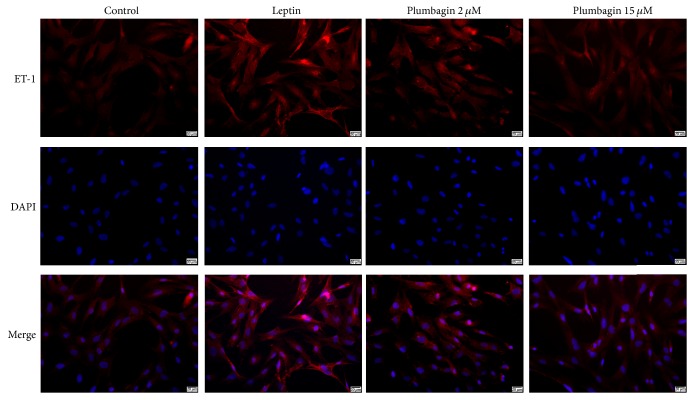
Plumbagin decreases the levels of ET-1, VEGF, LN, and type IV collagen, as determined by immunofluorescence assay. LSECs were treated with plumbagin after stimulation with leptin. Cells treated with vehicle were used as controls. ET-1 (red) were widely observed in cytoplasm of LSECs. The proteins were immunostained using cytokeratin-19 antibody and are shown in red. Nuclei were stained using DAPI (blue). Scale bar, 20 *μ*m.

**Figure 4:**
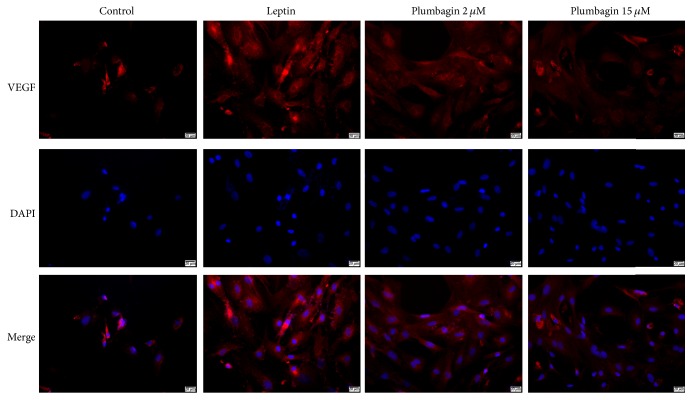
Plumbagin decreases the levels of VEGF, as determined by immunofluorescence assay. VEGF (red) were widely observed in cytoplasm of LSECs.

**Figure 5:**
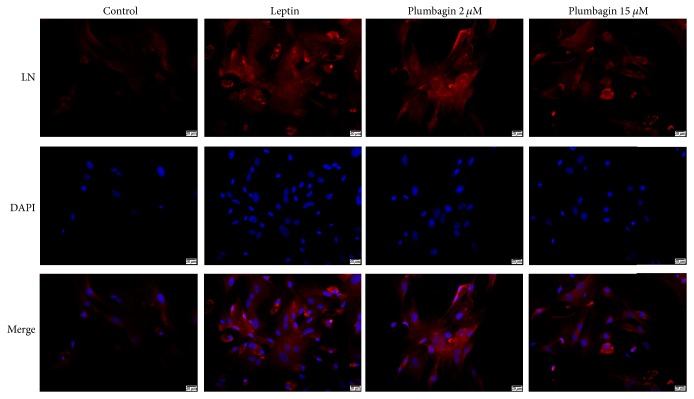
Plumbagin decreases the levels of LN, as determined by immunofluorescence assay. LN (red) accumulated on cytomembrane of LSECs.

**Figure 6:**
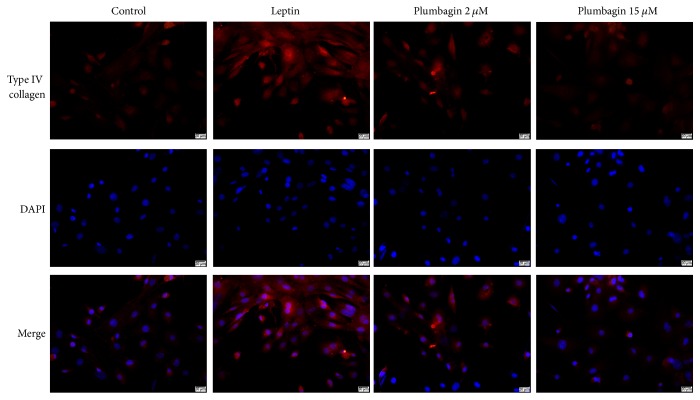
Plumbagin decreases the levels of type IV collagen, as determined by immunofluorescence assay. Type IV collagen (red) accumulated on cytomembrane of LSECs.

To detect the expression of ET-1, VEGF, LN, and type IV collagen in LSECs, immunofluorescence was used. ET-1 (Figure 3) and VEGF (Figure 4) were deposited in the cytoplasm of LSECs (Figure 5), while LN and type IV collagen were deposited on the cytomembrane of LSECs (Figures 5 and 6). ET-1, VEGF, LN, and type IV collagen from the leptin-stimulated LSECs were markedly deposited in LSECs compared with vehicle control. However, plumbagin decreased accumulation of these cytokines, including ET-1, VEGF, LN, and type IV collagen. Plumbagin with 15 *μ*mol/L administration has better effects observed upon reversing deposition of those proteins produced by leptin-stimulated LSECs.

### 3.4. Plumbagin Inhibits the Expression of ET-1, VEGF, LN, and Type IV Collagen in the Leptin-Stimulated LSECs

**Figure 7:**
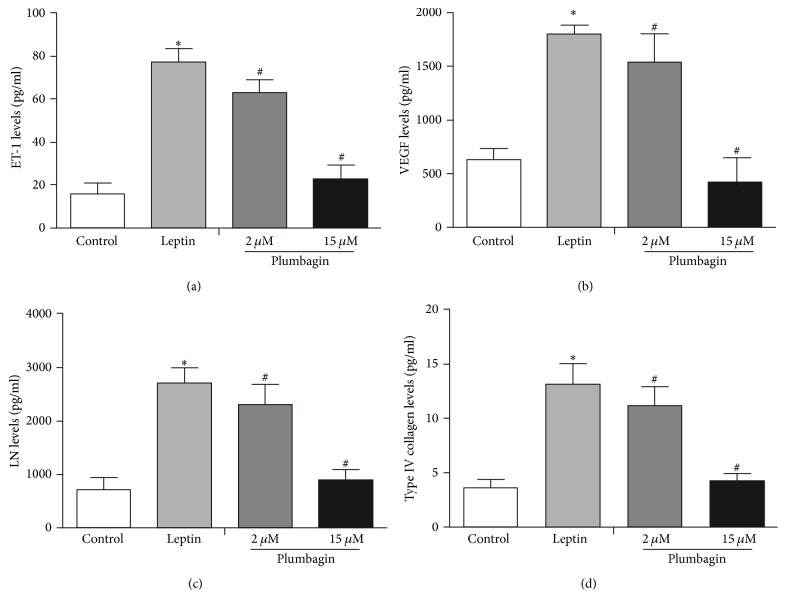
Protein expression by ELISA after treatment with plumbagin. (a) Levels of ET-1, (b) levels of VEGF, (c) levels of LN, and (d) levels of type IV collagen. The data are analyzed using ANOVA with Bonferroni posttests through SPSS 16.0 software. The results are displayed in a bar graph as the mean ± SD. ^*∗*^*P* < 0.05 versus control, ^#^*P* < 0.05 versus leptin.

The ELISA results showed that leptin-induced LSECs produced large amounts of ET-1, VEGF, LN, and type IV collagen compared with the levels in the vehicle control (*P* < 0.05). Nevertheless, plumbagin-administered LSECs presented with reductions of ET-1, VEGF, LN, and type IV collagen expressions (*P* < 0.05) (Figure 7).

### 3.5. Plumbagin Downregulates Expression of ET-1, VEGF, LN, and Type IV Collagen mRNA

**Figure 8:**
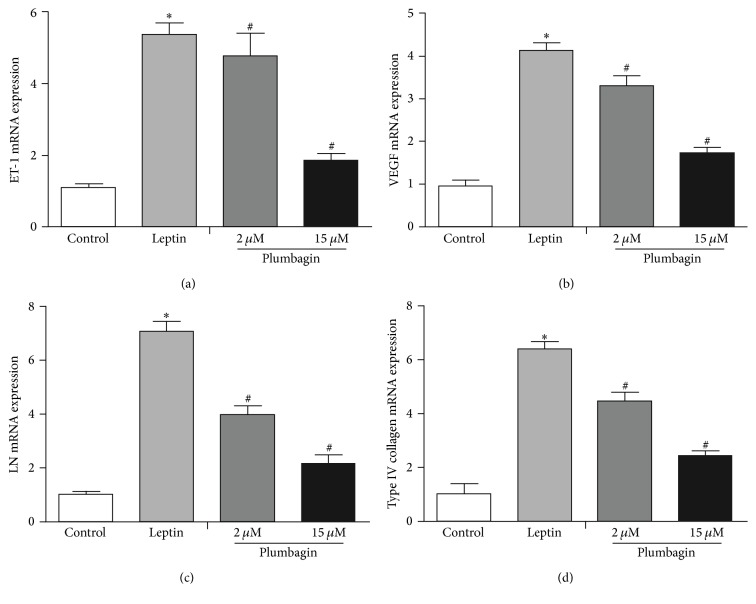
qPCR analyses for ET-1, VEGF, LN, and type IV collagen mRNA in LSECs. (a) Levels of ET-1 mRNA, (b) levels of VEGF mRNA, (c) levels of LN mRNA, and (d) levels of type IV collagen mRNA. The data are analyzed using ANOVA with Bonferroni posttests through SPSS 16.0 software. The results are displayed in a bar graph as the mean ± SD. ^*∗*^*P* < 0.05 versus control, ^#^*P* < 0.05 versus leptin.

We assessed ET-1, VEGF, LN, and type IV collagen mRNA expression in LSECs by qPCR and found that LSECs from the vehicle control showed lower levels. Conversely, leptin stimulation in LSECs resulted in a considerable upregulation of levels of ET-1, VEGF, LN, and type IV collagen mRNA compared to levels in the vehicle control. After plumbagin administration, LSECs expressed notably lower levels of ET-1, VEGF, LN, and type IV collagen mRNA than the plumbagin-untreated group. These effects of plumbagin were also dose dependent (Figure 8).

## 4. Discussion

Uncontrolled chronic liver injuries result in liver fibrosis, which is characterized by an enhanced extracellular matrix (ECM) [23]. Therefore, liver fibrosis is related to the ECM. It is well known that liver fibrosis can eventually lead to the development of cirrhosis and liver failure [24]. Liver fibrosis is a common disease, and effective treatment is seriously needed [25]. Large studies have shown that liver fibrosis can be controlled and reversed [26]. Our previous study [27] has revealed that plumbagin has an antifibrotic effect on liver fibrosis in vivo. Our present data indicate that plumbagin returns sinusoidal capillarization in vitro.

Sinusoidal capillarization is characterized by the formation of a basement membrane and defenestration of LSEC. It precedes hepatic fibrogenesis and promotes fibrotic processes [9]. Our present data show that there is no basement membrane in normal LSECs due to large amounts of open fenestrae whose diameters are 1–0.1 *μ*m. Nevertheless, loss of fenestrae due to LN and type IV collagen deposition resulted in continuous basement membrane formation in leptin-stimulated LSECs. Leptin induces LSEC activation. In contrast, plumbagin reverses this pathology in leptin-stimulated LSEC. In this study, we also found that leptin enhances ET-1, VEGF, LN, and type IV collagen expression, and all of these have been used to induce liver fibrosis. We found that plumbagin downregulates the expression levels of these cytokines and inhibits the expression of ET-1, VEGF, LN, and type IV collagen mRNA. Interestingly, these effects presented in a dose-dependent manner, with the higher dose of plumbagin eliciting better effects. Combined with the data above, we demonstrated that plumbagin contributes to the reversal of pathological changes seen in leptin-stimulated LSECs such as the capillarization of hepatic sinusoids via downregulating ET-1, VEGF, LN, and type IV collagen expression.

ET-1 is a potent regulator of vascular function produced in the liver via LSEC. In CCL_4_-induced mice liver fibrosis, ET-1 gene expression is increased [13]. ET-1 binds to two types of G protein-coupled receptors: endothelin type A receptor (ETAR) and type B receptor (ETBR). Transduction of the large biological effects of ET-1 is caused by ETAR. ETBR plays a central role in receptor clearance. Several vasoactive peptides and growth factors may cause their effects via ET-1 [28].

Endothelial NO synthase (eNOS) can be stimulated by ETBR to enhance NO production [29]. Failure of ETBR-activated eNOS causes disturbance of the hepatic microcirculation. Overexpression of ET-1 in LSEC accelerates the production of inflammatory mediators [17]. LSECs secrete and synthesize ET-1 [16], which induces quiescent hepatic stellate cell (HSC) and LSEC constriction [30]. The action of ET-1 on HSCs results in sinusoidal narrowing, which may promote the disturbance of blood circulation in hepatic sinusoid and endotoxin-induced liver injury. HSC is a major cell type contributing to liver fibrosis via inflammation, cytokines, chemokines, and vasoactive mediators [31, 32]. All together, they promote loss of open fenestrae on LSEC as well as the formation of a continuous basement membrane (capillarization). This suggests that ET-1 may facilitate capillarization. Previous studies have illustrated that overexpression of ET-1 produced by LSEC increases intrahepatic resistance and benefits portal hypertension [33]. Increased serum ET-1 concentrations are found in all stages of liver fibrosis [34]. Upregulation of ET-1, which induces myofibroblasts to contract, is responsible for hepatic wound healing and fibrosis [16]. ET-1 may have a profibrotic effect on liver fibrosis. This study indicated the roles of ET-1 on LSECs. In normal LSECs, there is less ET-1, while, in leptin-stimulated LSECs, it excessively deposits in the cytoplasm of LSECs. ET-1 may also participate in the pathological changes seen in LSECs such as defenestration and the formation of a basement membrane. Plumbagin inhibits liver fibrosis by decreasing the expression of epidermal growth factor receptor (EGFR) and transcription factor 3 (STAT3) in the liver [35]. Interestingly, the antifibrotic effects of plumbagin may also be related to other novel signaling pathways. Our data demonstrate that plumbagin ameliorates dysfunction of LSECs stimulated by leptin via downregulating ET-1, which are associated with capillarization of hepatic sinusoids and fibrosis return.

VEGF, a key regulator of angiogenesis, mediates proliferation and migration of LSECs during the wound healing process [36]. Experimental evidence has suggested that, during liver fibrosis and cirrhosis, the process of angiogenesis is associated with the progress of hepatic fibrogenesis [19]. Interestingly, pathological angiogenesis contributes to HSC activation causing liver fibrosis. In the liver, angiogenesis and fibrogenesis are induced by VEGF, which increases in the liver of cirrhotic rats and cirrhosis patients [37]. Notably, the roles of VEGF signaling in liver fibrogenesis have been explored via numerous studies [38]. Inhibiting VEGF activity markedly alleviates angiogenic and fibrogenic reactions [39]. The excessive expression of VEGF enhances liver fibrosis through the secretion of liver collagen [40]. Notably, the growth of LSECs in early liver fibrosis is promoted through VEGF [41]. LSECs are considered as major promoter of angiogenesis [38]. However, balance of intercellular contacting in adherens junctions is blocked and vascular permeability is enhanced by VEGF, resulting in the formation of a basement membrane in the Disse's space and phenotype changes in LSECs [42]. This suggests that VEGF causes dysfunction and structural changes in LSECs and thus promotes hepatic sinusoidal microcirculation disturbances and capillarization. In this study, we found that VEGF is markedly increased and accumulates in the cytoplasm of LSECs. In addition, there is upregulation of VEGF mRNA and protein levels in leptin-stimulated LSECs. However, plumbagin administration reverses these pathological changes. The drug may mitigate defenestration of LSEC via downregulating VEGF and VEGF mRNA.

LN is shown to be significantly increased in fibrotic rats and chronic hepatitis patients [43–45]. LN maintains the structure of basement membrane and regulates a large range of cellular functions, including adhesion, migration, growth, differentiation, and apoptosis. LN plays a crucial role in regulating the function of LSECs in sinusoidal reconstruction. During liver regeneration, LSECs may upregulate LN. LN is also associated with structural changes in LSECs, especially the open fenestrations of LSECs [46, 47]. Overexpression of LN in LSECs promotes capillarization of hepatic sinusoids. LN is found in the space of Disse between hepatocytes and LSECs and is related to the defenestration of LSEC as well as morphological changes in LSECs during liver fibrosis [48].

Excessive depositions of ECM proteins, containing types I, III, and IV collagens, LN, and fibronectin in the liver, are features of hepatic fibrogenesis [19]. Both LN and type IV collagen are primary ingredients of the basement membrane. Activation of LSECs stimulates LN and type IV collagen production [49]. Type IV collagen and LN, which are produced by LSECs, deposit in the sinusoids in liver fibrosis [50]. Hepatic sinusoidal blood flow is regulated via LSECs [51]. As fibrosis progresses, type IV collagen and LN are increased in the space of Disse and contribute to the formation of a basement membrane. Moreover, this process promotes the defenestration of LSECs and thus disturbs the microcirculation in the liver (termed “capillarization”), suggesting that type IV collagen and LN play pivotal roles in the defenestration of LSECs [12]. Our data show that LN and type IV collagen are increased and deposited in the cytomembrane of LSECs stimulated by leptin. Additionally, overexpression of LN and type IV collagen mRNA is also detected on LSECs stimulated by leptin. Plumbagin diminishes LN and type IV collagen and downregulates expression of LN and type IV collagen mRNA in LSECs stimulated by leptin. This suggests that plumbagin alleviates capillarization of hepatic sinusoids via downregulation of LN and type IV collagen.

In conclusion, our results reveal that leptin-stimulated LSECs present basement membrane formation due to defenestration. Upregulation of profibrogenic factors, including ET-1, VEGF, and basement membrane components (LN and type IV collagen), also occurs in the leptin-stimulated LSECs during capillarization of hepatic sinusoids. Plumbagin mitigates capillarization of hepatic sinusoids via downregulating ET-1, VEGF, LN, and type IV collagen, and this provides new insight into the signaling pathways that plumbagin can affect in LSECs. Moreover, a new therapeutic strategy for liver fibrosis may be further explored based on this study.

## 5. Conclusion

Plumbagin ameliorates capillarization of hepatic sinusoids by downregulating ET-1, VEGF, LN, and type IV collagen in vitro.
